# Clinical Efficacy of Immune Checkpoint Inhibitors in Patients With Advanced Malignant Peritoneal Mesothelioma

**DOI:** 10.1001/jamanetworkopen.2021.19934

**Published:** 2021-08-06

**Authors:** Kanwal Raghav, Suyu Liu, Michael Overman, Ajaykumar Morani, Anneleis Willette, Keith Fournier, Gauri Varadhachary

**Affiliations:** 1 Department of Gastrointestinal Medical Oncology, The University of Texas MD Anderson Cancer Center, Houston; 2 Department of Biostatistics, The University of Texas MD Anderson Cancer Center, Houston; 3 Department of Diagnostic Imaging, The University of Texas MD Anderson Cancer Center, Houston; 4 Department of Surgical Oncology, The University of Texas MD Anderson Cancer Center, Houston

## Abstract

This cohort study examines the clinical efficacy of immune checkpoint inhibitors (ICIs) in patients with advanced malignant peritoneal mesothelioma.

## Introduction

Malignant peritoneal mesothelioma (MPeM) is a rare malignant entity with an annual incidence of 0.10 cases per 100 000 population in the US.^1^ Patients with advanced, unresectable disease have limited systemic treatment options and poor survival.^1^ There is no standard or approved treatment beyond first-line platinum-pemetrexed therapy, and there is a critical unmet need for treatment for patients with this rare disease. Immune checkpoint inhibitors (ICIs) are likely to be effective in treating MPeM as a result of its proinflammatory microenvironment and abundant programmed cell death–ligand 1 expression.^2^ However, exclusion of patients with MPeM from mesothelioma clinical trials with ICIs has created a knowledge gap regarding their efficacy in MPeM. Because of promising results seen in malignant pleural mesothelioma (MPM), ICIs have been used off-label (in the absence of a clinical trial option) at MD Anderson Cancer Center.^2,3,4^ Given the lack of prospective data, we aimed to define the clinical efficacy of ICIs in patients with MPeM.

## Methods

We performed a cohort study of all consecutive patients with advanced MPeM treated with ICIs between January 2016 and December 2020 under an MD Anderson Cancer Center institutional review board–approved protocol. Informed consent was waived because the data were deidentified and the study was considered to pose minimal risk to participants, in accordance with 45 CFR §46 (see the study schema in eFigure in the Supplement). Clinical, treatment, and outcomes data were collected using electronic medical records. The primary end point was objective response rate (ORR) per Response Evaluation Criteria in Solid Tumors version 1.1. Secondary end points were progression-free survival (PFS), time to treatment failure, and overall survival (OS). The Clopper-Pearson method was used to calculate exact 95% CIs for proportions. The Fisher exact test (2-sided) was used for comparisons between groups, with significance set at *P* < .05. Statistical analyses were performed using SPSS statistical software version 25.0.0.1 (IBM) and Prism software version 8.00 (GraphPad). See eMethods in the Supplement for additional details.

## Results

Baseline characteristics of 29 patients (median [range], 60 [38-77] years; 15 women [52%]) with MPeM treated with ICIs (20 with dual-inhibition [nivolumab plus ipilimumab] and 9 with single-agent ICIs) are shown in the Table. Among evaluable patients, the ORR was 19.2% (5 of 26 patients; 95% CI, 6.6%-39.4%); the response was ongoing in 1 of 5 patients (20.0%) at data cutoff (Figure, A). No significant difference in ORR was seen between key clinical subgroups, including dual-agent and single-agent ICI (Table). The disease-control rate was 65.4% (95% CI, 44.0%-83.0%). At data cutoff, the median follow-up was 9.8 months (95% CI, 6.5-18.7 months), and 24 patients had discontinued ICI therapy (20 experienced progressive disease, 3 died, and 1 experienced toxic effects). The median duration of PFS was 5.5 months (95% CI, 3.4-9.2 months), and the 1-year PFS rate was 14% (95% CI, 4%-31%) (Figure, B). The time to treatment failure rate at 1 year was 13% (95% CI, 3%-30%). The median duration of OS was 19.1 months (95% CI, 7.4-43.2 months), and the 1-year OS rate was 68% (95% CI, 45%-83%) (Figure, D). Treatment was well tolerated (Figure, C).

**Table.** Baseline Characteristics of Patients With Malignant Peritoneal Mesothelioma Treated With ICIs^a^

Characteristic	Patients, No. (%) (N = 29)	ORR, % (95% CI)^b^	*P* value^c^
Age at enrollment, median (range), y	60 (38-77)	NA	NA
<60	13 (45)	33 (10-65)	.15
≥60	16 (55)	7 (0-34)	
Sex			
Female	15 (52)	23 (5-54)	.99
Male	14 (48)	15 (2-45)	
Performance status (Eastern Cooperative Oncology Group)			
0 or 1	23 (79)	22 (8-45)	.55
2	6 (21)	0 (0-60)	
Tumor histological profile			
Epithelioid	25 (86)	22 (7-44)	.99
Biphasic or sarcomatoid	4 (14)	0 (0-71)	
Prior asbestos exposure^d^			
Yes	4 (15)	0 (0-71)	.99
No	23 (85)	23 (8-45)	
Time from diagnosis to therapy, median (range), y	1.8 (0.2-10.9)	NA	
<1	10 (34)	25 (3-65)	.99
≥1	19 (66)	18 (4-43)	
Prior cytoreductive surgery			
Yes	15 (52)	17 (2-48)	.99
No	14 (48)	21 (5-51)	
Prior lines of anticancer therapy, No.			
1	24 (83)	18 (5-40)	.99
2 or 3	5 (17)	25 (1-81)	
Response to prior platinum-pemetrexed^e^			
Regression	11 (38)	20 (3-56)	.99
Stability or progression	18 (45)	19 (4-46)	
ICI used			
Nivolumab plus ipilimumab	20 (69)	18 (4-43)	.99
Single-agent ICI^f^	9 (31)	22 (3-60)	
Next-generation sequencing^g^	17 (59)		
*BAP1* variants (loss)	3 (18)	33 (1-91)	NA
*NF2* variants	5 (29)	40 (5-85)	

Abbreviations: ICI, immune checkpoint inhibitor; NA, not applicable; ORR, objective response rate.

^a^ Response to immunotherapy was assessed as per Response Evaluation Criteria in Solid Tumors version 1.1 in evaluable population (measurable disease and ≥1 restaging scan).

^b^ The ORR was defined as proportion of patients with a complete or partial response. The 95% CIs were computed by the Clopper and Pearson method for proportions.

^c^ The Fisher exact test was used for comparisons between groups.

^d^ Data are missing. Proportions are calculated from patients with available data.

^e^ Response to prior platinum-pemetrexed chemotherapy was assessed as per radiologist and treating physician discretion and reported as either disease regression, stability, or progression.

^f^ Atezolizumab, nivolumab, and pembrolizumab were given to 2, 4, and 3 patients, respectively.

^g^ Next-generation sequencing was performed using OncoMine/FoundationOne/CARIS profiling. Programmed cell death–ligand 1 expression was available for 4 cases (range, 0%-10%). Tumor mutation burden was available for 4 patients (3-8 mutations/megabase).

**Figure.**
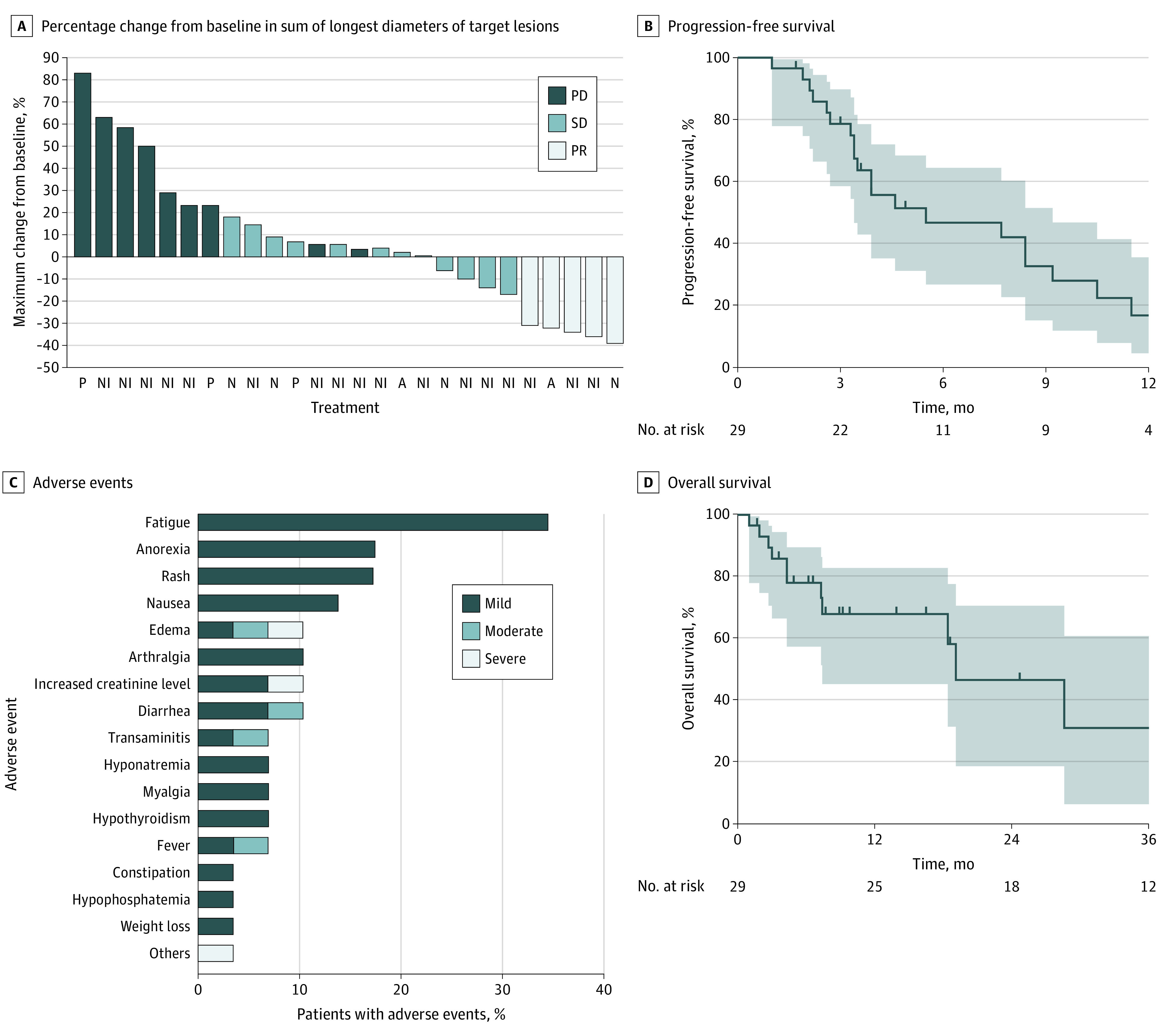
Tumor Response, Survival Outcomes, and Adverse Events Associated With Immune Checkpoint Inhibitor (ICI) Treatment Among Patients With Malignant Peritoneal Mesothelioma A, Waterfall plot shows the maximum percentage change from baseline in the sum of the longest diameters (short axis in case of lymph nodes) of target lesions in 26 evaluable patients receiving atezolizumab (A), nivolumab (N), nivolumab plus ipilimumab (NI), and pembrolizumab (P). Tumor measurements and response assessments (progressive disease [PD], partial response [PR], and stable disease [SD]) were performed according to Response Evaluation Criteria in Solid Tumors version 1.1. B and D, KapIan-Meier curves show progression-free survival (B, measured from treatment initiation to disease progression or death) and overall survival (D, measured from treatment initiation to death) for 29 patients enrolled in the study at the time of data cutoff. Solid lines denote median survival rates, shaded areas denote 95% CIs, and vertical tick marks denote data censoring (ie, patient death). C, Graph shows proportion of adverse events (mild, moderate, and severe) seen in study cohort as annotated in electronic medical records. In total, 19 patients (65.5%) experienced any adverse events, and 5 patients (17.2%) had moderate or severe adverse events.

## Discussion

To our knowledge, in this cohort study, we report the first real-world evidence regarding clinical outcomes for a cohort of patients with advanced MPeM receiving ICIs. We observed encouraging clinical activity, with an ORR of 19.2% and a median PFS of 5.5 months. Disease response was seen regardless of response to prior platinum-pemetrexed chemotherapy. No significant difference in ORR was seen between key clinical subgroups, including dual-agent and single-agent ICI. Therapy also appeared to be well tolerated; only 1 patient had treatment discontinuation associated with toxic effects. This efficacy seems comparable to activity of ICIs in MPM.^3,4^ However, caution is needed when extrapolating data from MPM to MPeM because these diseases appear to be molecularly and immunologically different (eg, MPeM has higher programmed cell death–ligand 1 expression than MPM).^2,5^ Results from a phase 2 study of atezolizumab and bevacizumab in MPeM showed very promising confirmed ORR of 40% with durable responses and notable 1-year PFS of 61% and OS of 85%.^6^

Although our study has some inherent limitations of a retrospective single-institution series, pending prospective trials, our results provide much-needed data supporting the role of ICIs in patients with this rare disease, who cannot participate in clinical trials and otherwise have no or limited treatment options. The study also demonstrates that the clinical benefit associated with conventional ICIs is restricted to a small subset of patients and argues that there is a critical need for dedicated trials and larger cohorts to define biomarkers of response or resistance, early referral to clinical trials, and novel combinatorial strategies to enhance responses and outcomes for patients with MPeM.

## Notes

Supplement. eMethods. **eFigure.** Schema for Selection of Cohort of Patients With Advanced Malignant Peritoneal Mesothelioma Treated With Immune Checkpoint Inhibitors (ICIs)
