# Supplementary material for: Clinical Efficacy of Immune Checkpoint Inhibitors in Patients With Advanced Malignant Peritoneal Mesothelioma
Source: JAMA Netw Open. 2021 Aug 6;4(8):e2119934. doi: 10.1001/jamanetworkopen.2021.19934 (PMC8346939; doi:10.1001/jamanetworkopen.2021.19934)
Supplement: Supplement. — eMethods. eFigure. Schema for Selection of Cohort of Patients With Advanced Malignant Peritoneal Mesothelioma Treated With Immune Checkpoint Inhibitors (ICIs) [file jamanetwopen-e2119934-s001.pdf]

Supplemental Online Content

Raghav K, Liu S, Overman M, et al. Clinical efficacy of immune checkpoint inhibitors in patients with advanced malignant peritoneal mesothelioma. *JAMA Netw Open*. 2021;4(8):e2119934. doi:10.1001/jamanetworkopen.2021.19934

eMethods.

eFigure. Schema for Selection of Cohort of Patients With Advanced Malignant Peritoneal Mesothelioma Treated With Immune Checkpoint Inhibitors (ICIs)

This supplemental material has been provided by the authors to give readers additional information about their work.

© 2021 Raghav K et al. *JAMA Network Open*.

eMethods

We performed a retrospective review of a cohort of 126 consecutive patients with malignant peritoneal mesothelioma (MPeM) evaluated at The University of Texas MD Anderson Cancer Center, Houston, TX, USA (MDACC) over a period of 5 years (January 2016 and December 2020). For the purpose of this study, we included patients who had received off-label treatment with one or more immune-checkpoint inhibitors (ICIs) as part of their clinical care for advanced disease after at least one line of systemic treatment with platinum-pemetrexed chemotherapy, the current standard of care for patients with MPeM. Patients who received ICI in first-line therapy or in combination with other targeted agents were excluded. Patients underwent treatment (recruitment and exposure) and follow-up and data were collected between January 2016 and December 2020. Data on patient and tumor characteristics, treatment and outcomes were extracted from electronic medical records. To limit selection and informational bias, we 1) carefully defined inclusion criteria prior to data collections, 2) included all consecutive patients during a defined study period, 3) used clear and homogeneous definitions of disease, variables and exposure, and 4) ensured that all data were collected and vetted by investigator in a similar way. Response was assessed using Response Evaluation Criteria in Solid Tumors version 1.1 (RECISTv1.1). The study was performed under a protocol approved by the MDACC institutional review board with waiver of written informed consent by patients. This retrospective cohort study follows the STROBE reporting guideline for observational studies.

Statistical methodology

The primary objective of this study was to determine the clinical efficacy of ICIs in advanced MPeM who have had prior systemic platinum-pemetrexed chemotherapy. The primary endpoint was objective response rate (ORR: proportion of patients achieving a complete or partial response (CR or PR), per RECISTv1.1 in evaluable patients (those with at least one restaging for comparison). Secondary endpoints were progression-free survival (PFS: defined as the time from initiation of treatment with ICI until tumor progression or death), time-to-treatment failure (TTF: defined as the time

© 2021 Raghav K et al. *JAMA Network Open.*

from initiation of treatment with ICI to treatment discontinuation for any reason, including progressive disease, toxicity and death) and overall survival (OS: defined as the time from initiation of treatment with ICI to the death from any cause). No formal sample size calculations were used and all patients during the study period who met eligibility criteria were included in the cohort. Standard descriptive statistics were used. Clopper and Pearson method was used to calculate exact 95% confidence intervals (95%CI) for proportions. Kaplan-Meier method was used to estimate time to event endpoints and patients were censored if event of interest had not occurred at date of last follow-up. Fisher's exact test was used for comparisons between groups. Statistical analyses were performed using SPSS 25.0.0.1 (SPSS, Chicago, IL, USA) and GraphPad Prism version 8.00 (GraphPad software, La Jolla, CA, USA).

eFigure. Schema for Selection of Cohort of Patients With Advanced Malignant Peritoneal Mesothelioma Treated With Immune Checkpoint Inhibitors (ICIs).

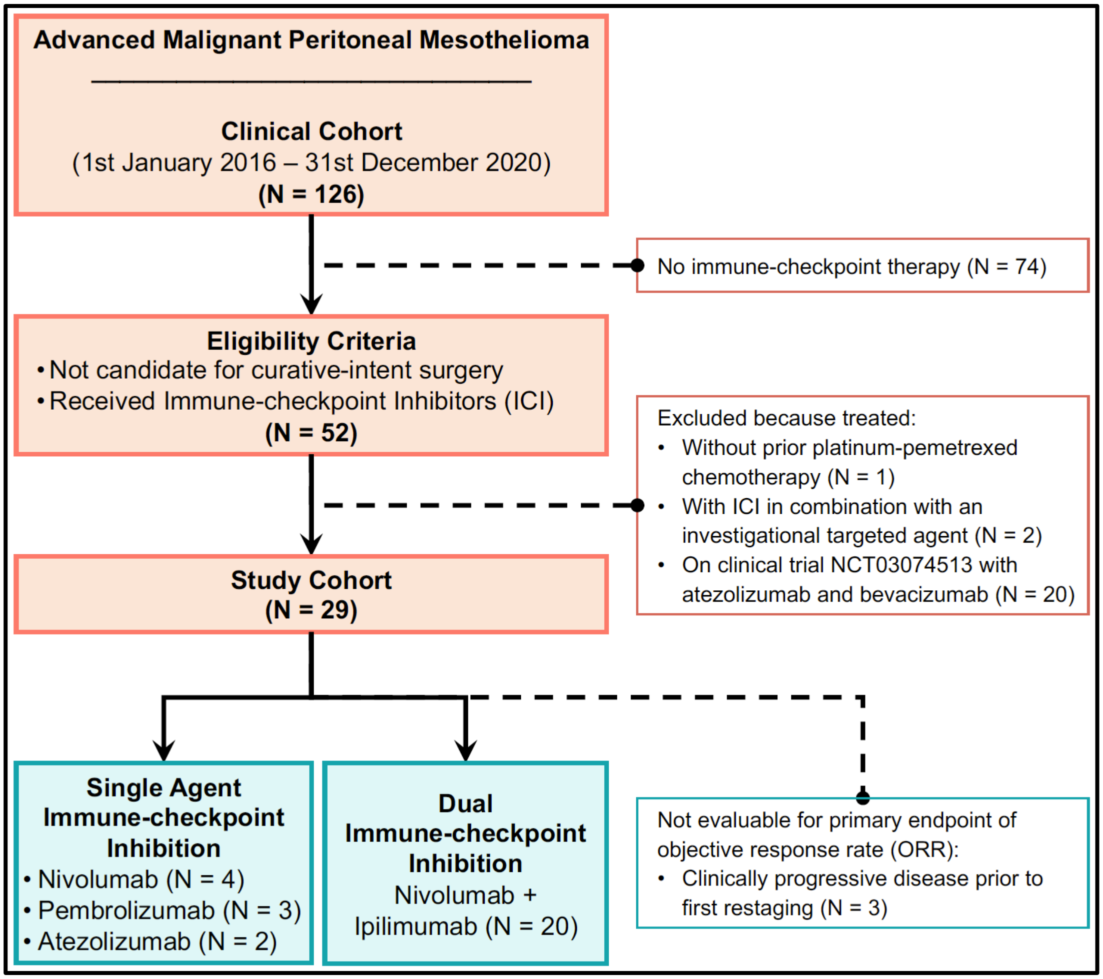

© 2021 Raghav K et al. *JAMA Network Open*.
